# Universal Identification of Pathogenic Viruses by Liquid Chromatography Coupled with Tandem Mass Spectrometry Proteotyping

**DOI:** 10.1016/j.mcpro.2024.100822

**Published:** 2024-07-30

**Authors:** Clément Lozano, Olivier Pible, Marine Eschlimann, Mathieu Giraud, Stéphanie Debroas, Jean-Charles Gaillard, Laurent Bellanger, Laurent Taysse, Jean Armengaud

**Affiliations:** 1 Département Médicaments et Technologies pour la Santé (DMTS), Université Paris-Saclay, CEA, INRAE, SPI, Bagnols-sur-Cèze, France; 2 Direction Générale de l’Armement Maîtrise NRBC, Vert-le-Petit, France

**Keywords:** virus, proteomics, diagnosis, mass spectrometry, proteotyping

## Abstract

Accurate and rapid identification of viruses is crucial for an effective medical diagnosis when dealing with infections. Conventional methods, including DNA amplification techniques or lateral-flow assays, are constrained to a specific set of targets to search for. In this study, we introduce a novel tandem mass spectrometry proteotyping-based method that offers a universal approach for the identification of pathogenic viruses and other components, eliminating the need for *a priori* knowledge of the sample composition. Our protocol relies on a time and cost-efficient peptide sample preparation, followed by an analysis with liquid chromatography coupled to high-resolution tandem mass spectrometry. As a proof of concept, we first assessed our method on publicly available shotgun proteomics datasets obtained from virus preparations and fecal samples of infected individuals. Successful virus identification was achieved with 53 public datasets, spanning 23 distinct viral species. Furthermore, we illustrated the method's capability to discriminate closely related viruses within the same sample, using alphaviruses as an example. The clinical applicability of our method was demonstrated by the accurate detection of the vaccinia virus in spiked saliva, a matrix of paramount clinical significance due to its non-invasive and easily obtainable nature. This innovative approach represents a significant advancement in pathogen detection and paves the way for enhanced diagnostic capabilities.

## Highlights

• Provides fully untargeted virus identification.
• Works on any matrices (stool, saliva, cell cultures).
• Provides the full picture of the organismal sample components.
• Discriminates closely related viruses within the same sample.

## Graphical Abstract

**Figure:**
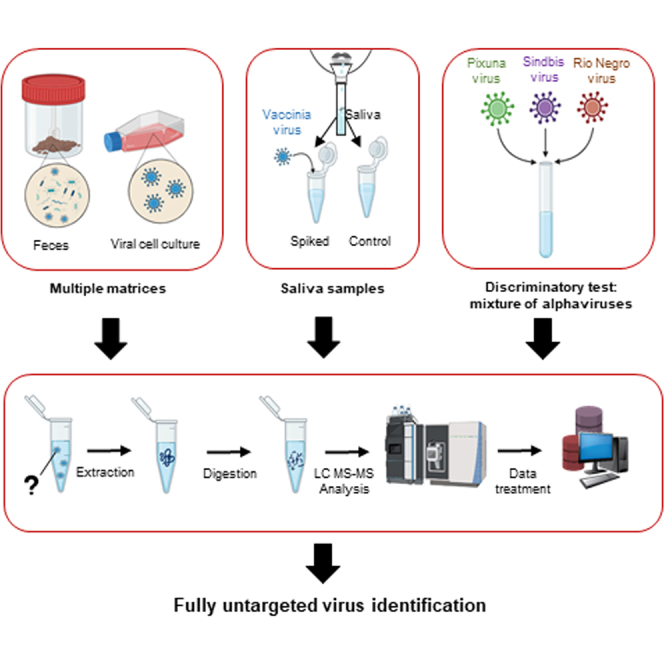

## In Brief

As illustrated by recent viral pandemics, rapid and accurate virus identification is of major interest. Here, we present a groundbreaking tandem mass spectrometry proteotyping method for rapid and universal virus identification, eliminating the need for predefined targets. Our approach, leveraging liquid chromatography and high-resolution tandem mass spectrometry, accurately identified 23 viral species across 53 datasets. Clinical applicability was demonstrated by detecting the vaccinia virus in saliva. This innovative method offers enhanced diagnostic capabilities, marking a significant advance in pathogen detection.

The development of new approaches for virus detection is of major societal interest as it may deliver more efficient diagnoses and improve countries’ resilience in major crises as suffered recently during the COVID-19 pandemic. Currently, polymerase chain reaction (PCR) is considered the gold standard for the identification of viral pathogens. However, several drawbacks can be listed: (i) it is limited to the targeted species, (ii) PCR may be sensitive to the emergence of variants, more specifically mutations occurring within the sequences corresponding to the primers and their surroundings, (iii) it is not adapted for the detection of newly engineered pathogens when CBRN-E threats are considered, and (iv) it cannot be used to monitor the advent of an unexpected new pathogen such as the "disease X" agent, a black swan risk considered by the WHO. Over the past decade, tandem mass spectrometry proteotyping rose as an interesting alternative to reinforce the array of molecular tools allowing rapid identification of microorganisms (1, 2, 3, 4). In complement to Matrix-Assisted Laser Desorption/Ionization Time-of-Flight, well implemented in clinical labs for the identification of bacterial species isolated after cultivation (5, 6), recent advances including spectral library-free proteotyping techniques and more discriminative approaches based on tandem mass spectrometry have further enriched the molecular detection toolbox (1).

Targeted MS-based approaches relying on Parallel Reaction Monitoring have been developed for virus detection, including SARS-CoV-2, Chikungunya, and Zika virus (7, 8, 9). While these techniques are specific and sensitive, they are confined to the detection of predefined organisms, thereby limiting their versatility. Alternatively, identification based on DNA shotgun sequencing has been shown as a promising approach but is still limited by long runtime (up to 3 days) and costs (10). In order to decrease the time to result, some authors have developed a real-time metagenomic-based diagnostic pipeline adapted to viruses (11). However, this concept does not provide a fully untargeted search as the database is limited to viruses and still requires around 24h before final results. Recently, a shotgun proteomics-based method coupled to a web application (Proteome2virus) has been designed to detect a panel of human pathogenic viruses (12). While this method represents a noteworthy improvement, it is still limited to the 46 viruses implemented in the database. Alternative tools such as TaxIt (2) and PepGM (13) are based on wider search space. Nonetheless, these tools may be susceptible to the utilization of non-exhaustive databases or specifically tailored to certain taxonomic kingdoms, hence limiting the detection possibilities. Moreover, their efficacy has not been tested on samples harboring complex microbial communities and eukaryotic signals.

Fully untargeted proteomics-based identification methodologies demonstrated their efficiency in the identification of numerous environmental and pathogenic microbial isolates (14, 15). Such an approach was identified from a few mg of an ancient relic a bacterium belonging to the *Actinomadura* genus, responsible for the chronic osteomyelitis of an individual who perished in 559 A.D (16). A similar strategy has been applied for virus identification (17) but remains to be validated with an array of viral pathogens. Saliva represents an interesting matrix for noninvasive diagnostic applications. The viral load in the saliva of positive patients has been previously documented for various viral species (18, 19, 20, 21) and appeared compatible with a tandem mass spectrometry assay.

Here, we documented a successful strategy for fully untargeted taxonomical identification of virus-containing samples *via* LC-MS/MS analysis. First, we illustrated the range of viruses identified from publicly available proteomic datasets. We showcased the discriminative power of the methodology by identifying three closely related alphaviruses within the same sample. Furthermore, we conducted a proof of concept experiment on saliva samples spiked with medically relevant quantities of the vaccinia virus.

## Experimental Procedures

### Vaccinia Virus Production

Vero E6 cells were cultivated in DMEM supplemented with 2.5% fetal calf serum and 0.5% penicillin-streptomycin at 37 °C under 9% CO_2_. Cells were infected with the vaccinia virus with a 0.01 multiplicity of infection. Viral particles were harvested 48 h post-infection *via* freeze-thaw cycles cell lysis and centrifugation at 2500*g*. The viral particles contained in the resulting supernatant were pelleted through a 36% (w/w) sucrose cushion at 110,000*g* for 2 h at 4 °C. Pelleted viruses were resuspended in 10 mM Tris-HCl and purified on a sucrose gradient *via* centrifugation at 17,000*g* for 45 min. Prior to inactivation at 70 °C for 10 min, titer was assessed *via* standard plaque assay resulting in a 9.10^7^ pfu/ml virus solution.

### Production of Alphaviruses

Vero cells were cultivated in M199 medium supplemented with 10% fetal bovine serum and 1% penicillin-streptomycin-amphotericin B at 37 °C under 5% CO_2_. Cells were infected with Pixuna virus, Rio Negro virus, or Sindbis virus with a multiplicity of infection of one in M199 medium supplemented with 2% fetal bovine serum and 1% penicillin-streptomycin-amphotericin B at 37 °C under 5% CO_2_. Viral particles were harvested 48 h post-infection and additional freeze-thaw cycles of cell lysis were conducted in order to release intracellular viral particles. Cell debris was eliminated by centrifugation at 1500*g*. The viral suspensions were titrated using the limit-dilution method and the Spearman-Kärber calculation method (22). The viral titers obtained were 1.10^9^ pfu/ml for the Pixuna virus, 7.5.10^8^ pfu/ml for the Rio Negro virus, and 2.4.10^8^ pfu/ml for the Sindbis virus. The virus particles were inactivated at 95 °C for 10 min. For obtaining a mixture of the three viruses, 1 ml of each virus solution were pooled with 2 ml of sterile water (5 ml final) before subsequent treatments.

### Saliva-Spiked Sample Preparation

Saliva from four healthy donors was collected. For each saliva sample, 100 μl was used as a negative control and 100 μl was spiked with purified virus to reach 5.10^6^ pfu/ml. A total of 50 μl of LDS buffer was added to reach the following concentrations: 26.5 mM Tris HCl, 35.25 mM Tris base, 0.5% LDS, 2.5% Glycerol, and 0.13 mM EDTA, and 5% β-mercaptoethanol. Samples were heated for 5 min at 99 °C in a thermomixer (Eppendorf) and sonicated for 5 min in an ultrasonic water bath (VWR ultrasonic cleaner). Biological material was transferred into 0.5 ml Screw Cap microtubes (Sarstedt) containing 50 mg of a custom-made bead mixture (23). Bead beating was performed with a Precellys Evolution instrument (Bertin Technologies) at 10,000 rpm for 10 cycles of 30 s, with 30 s of pause between each cycle. Proteins were centrifuged at 16,000*g* for 1 min. The resulting supernatants were transferred to new microcentrifuge tubes before incubation at 99 °C for 5 min for full denaturation.

### Protein Proteolysis

Proteins were digested *via* an SP3 procedure as previously described (24). Briefly, a 1:1 mix of hydrophilic (Ref. GE45152105050250) and hydrophobic (Ref. GE65152105050250) SpeedBeads magnetic beads (Merck) was prepared at 50 mg/ml and stored at 4 °C until use. 20 μL of protein solution were subjected to reduction and alkylation with 5 mM DTT and 15 mM iodoacetamide for 10 min in the dark at room temperature. A total of 200 μg of the bead mixture (4 μl) was added to the protein solution. Proteins were aggregated on beads by adding 200 μl of 85% acetonitrile/15% water solution. Bead–protein complexes were trapped using a magnetic stand 96 (Invitrogen) and the supernatant was discarded. Proteins were washed twice with 200 μl of 70% ethanol and once with 180 μl acetonitrile. Proteins were digested at 50 °C for 30 min with 30 μl of digestion buffer containing 0.1 μg of Trypsin Gold (Promega) in 50 mM NH_4_HCO_3_. Beads were trapped as described above and the resulting peptides were transferred into vials and acidified with 0.5% trifluoroacetic acid before nanoLC-MS/MS analysis.

### NanoLC MS/MS Analysis

Two hundred nanograms of peptides were analyzed with an Orbitrap Exploris 480 (Thermo Scientific) tandem mass spectrometer coupled to a Vanquish Neo UHPLC system (Thermo Scientific). Peptides were desalted on a reverse phase PepMap 100 C18 trapping column (5 µm, 100 Å, 300 µm × 5 mm) and separated on a 50 cm EasySpray column (2 µm, 100 Å, 75 µm × 500 mm C18 2 µm, Thermo Scientific) at a flow rate of 0.25 μl/min using a 30 min gradient (5%–25% B from 0 to 30 min, and 25%–40% B from 30 to 35 min) of mobile phase A (0.1% HCOOH/100% H_2_O) and phase B (0.1% HCOOH/100% CH_3_CN). The mass spectrometer operated in data-dependant acquisition mode with full-scan mass spectra acquired from *m/z* 350 to 1500. Only peptides with two or three positive charges were selected for fragmentation in a Top10 strategy with a dynamic exclusion time of 5 s and an *m/z* isolation window of 1.1.

### MS/MS Spectra Analysis

**Fig. 1:**
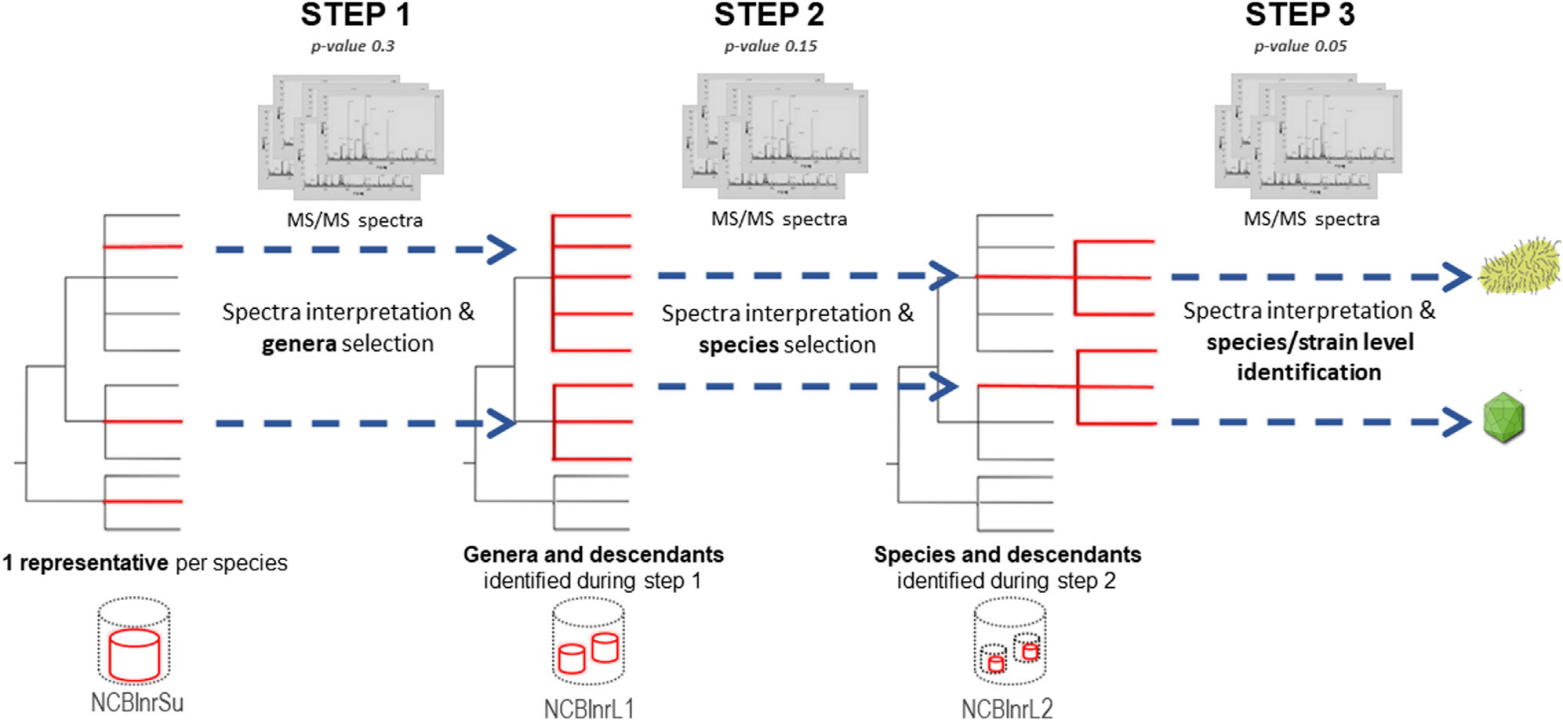
**Database construction along the cascade search strategy for *a priori* free proteotyping-based organism identification**. Branches of the phylogenetic tree highlighted in red show the taxa included in the database at each of the three steps of the cascade search. During the first step, a subset of NCBInr harboring one representative per species (NCBInrS) is queried. Based on the results of this first iteration, the database used for the second step is constructed, including all identified genera and their descendants. Similarly, the database used for the third step is constructed based on the species detected during step 2, and their descendants. Taxa that are identified and used to construct the database of the next step are indicated by *blue arrows*.

Taxonomical identification was performed using the Mascot version 2.6.1 (Matrix Science) search engine and a cascade search as follows (Fig. 1). In the first round, the 10,000 best MS/MS spectra were selected and queried against a subset of the NCBInr database, referred to as NCBInrS. This subset comprises one representative per species and includes 94,176,939 protein sequence entries totaling 39,636,215,241 amino acids and corresponding to 50,995 organisms (494 Archaea, 2231 Eukaryota, 12,047 Bacteria, and 36,223 Viruses). During the second round, all MS/MS spectra were used for a Mascot query against a database reduced to the genera identified during the first round, and all their descendants. The last round consisted of the search of all MS/MS spectra against a database reduced to the species identified during the second search. Peptides were validated using a *p*-value threshold set at 0.3, 0.15, and 0.05 for steps 1, 2, and 3, respectively, as previously recommended (25). The search engine was configured as follows: peptide tolerance set at 3 ppm (step 1) or 5 ppm (steps 2 and 3), 0.02 Da MS/MS fragment tolerance, 2+ or 3+ peptide charges, a maximum of one missed cleavage during the first round, and two missed cleavages for round 2 and 3, carbamidomethylation of cysteine as fixed modification, oxidation of methionine as variable modification, and trypsin as proteolytic enzyme. The list of identified peptides and their respective sequence, precursor charge, mass-to-charge ratio, all modifications observed, and their ion scores are listed in Supplemental Table S1.

### Experimental Design and Statistical Rationale

The use of four different saliva donors ensures that the findings are not donor-specific and can be generalized across different biological variations. Specificity was assessed by analyzing control samples where no virus is expected and ensuring no false positives were detected.

## Results

### Virus Identification From Multiple LC-MS/MS Public Datasets

We randomly selected a series of public datasets from studies where a broad range of viral species have been studied by proteomics (Table 1). Data were acquired either from purified viral particles from infected cell cultures or fecal samples from healthy or infected patients. These datasets were acquired with various LC-MS/MS settings, with a wide range of Orbitrap instruments (including Q-exactive, Q-exactive plus, LTQ orbitrap discovery, LTQ orbitrap XL and Orbitrap Eclipse) and separation gradients (ranging from 60 to 240 min).

**Table 1:** Summary of virus detection results obtained from public datasets

Public repository identifier and reference	File name on public repositories	Identified species	TSM	SpePep	Estimated biomass %
PXD036663 Balvers et al. (2023) (12)	201108OEc1_LD-2068-TNO-6B_018	Severe acute respiratory syndrome-related coronavirus	27	23	1.16
	201108OEc1_LD-2068-TNO-6A_017	Severe acute respiratory syndrome-related coronavirus	78	51	1.23
	200417OEc1_LD-1999-TNO-15-1Aa_017	Severe acute respiratory syndrome-related coronavirus	99	42	1.45
	211115OEc1_AP-2216-BM1650-010x--DDA90_009	ND	0	0	NA
	211115OEc1_AP-2216-BM1650-100x--DDA90_011	Human coronavirus 229E	62	32	0.72
	211115OEc1_AP-2216-BM1649-100x--DDA90_007	Betacoronavirus 1	125	50	1.01
	211115OEc1_AP-2216-BM1649-010x--DDA90_005	Betacoronavirus 1	144	53	1.06
	220311OEc1_AP-2231-s6B-10uL--DDA90_021	Influenza A virus	311	243	1.40
	220311OEc1_AP-2231-s6C-10uL--DDA90_012	Influenza A virus	77	83	2.47
	220311OEc1_AP-2231-s5B-10uL--DDA90_018	Influenza A virus	556	456	3.50
	220311OEc1_AP-2231-s5C-10uL--DDA90_009	Influenza A virus	120	134	6.14
	220311OEc1_AP-2231-s2--03uL--DDA90_039	Negative controls	0	0	NA
	220311OEc1_AP-2231-s3--01uL--DDA90_051	Negative controls	0	0	NA
	220311OEc1_AP-2231-s4B-10uL--DDA90_015	Human metapneumovirus	197	113	0.68
	211115OEc1_AP-2216-BM1453-010x--DDA90_C023	Human orthopneumovirus	209	155	0.81
	211115OEc1_AP-2216-BM1453-100x--DDA90_C019	Respiratory syncytial virus	63	44	0.93
	220311OEc1_AP-2231-s4C-10uL--DDA90_006	Human metapneumovirus	47	39	1.04
	220311OEc1_AP-2231-s7B-01uL--DDA90_042	Respiratory syncytial virus	207	114	1.36
	220328OEc1_AP-2231-s7C-01uL--DDA90_014	Human orthopneumovirus	71	69	1.55
	**150924QXc1_RB-0965-150922-003_009**	ND	0	0	
	**150924QXc1_RB-0965-150922-001_002**	ND	0	0	NA
	**150924QXc1_RB-0965-150922-008_019**	Norwalk virus	7	4	0.12
	**150924QXc1_RB-0965-150922-002_007**	Norwalk virus	17	10	0.35
	**150924QXc1_RB-0965-150922-007_017**	Rotavirus A	48	31	2.51
	**150924QXc1_RB-0965-150922-004_011**	Rotavirus A	836	191	11.18
	**150924QXc1_RB-0965-150922-006_015**	Human mastadenovirus F	300	47	3.86
	**150924QXc1_RB-0965-150922-009_021**	Human mastadenovirus F	865	61	18.75
PXD003013 Doellinger et al. (2015) (41)	CPXV_Kre_2	Cowpox virus	3463	61	43.72
	CPXV_Kre_3	Cowpox virus	3639	59	44.36
	VACV_Cop_1	Cowpox virus	3726	40	52.47
	VACV_NY_1	Vaccinia virus	2543	55	28.71
	VACV_WR_2	Vaccinia virus	3392	39	48.21
	VACV_WR_1	Vaccinia virus	3452	39	48.77
	VACV_WR_3	Vaccinia virus	3462	156	49.37
PXD000591 Benevento et al. (2014) (42)	HAdV_1h_Qexactive_2	Human mastadenovirus C	1631	28	79.83
	HAdV_1h_Qexactive_3	Human mastadenovirus C	1929	33	83.76
	HAdV_1h_Qexactive	Human mastadenovirus C	1353	22	84.40
	HAdV_90m_Qexactive	Human mastadenovirus C	1072	23	77.07
	HAdV_90m_Qexactive_2	Human mastadenovirus C	1203	22	80.58
MSV000078740 Hutchinson et al. (2014) (43)	B111206_009	Influenza A virus	6	16	21.43
	C130701_010	Influenza A virus	1185	387	47.23
	B120210_001	Influenza A virus	22	33	57.89
	20170120_Q1_TC_colQ1-36_Legros_Pf13-24-2	ND	0	0	NA
	20170120_Q1_TC_colQ1-36_Legros_Pf13-48-2	Zika virus	78	78	0.24
PXD015316 Meignié et al. (2021) (44)	20170912_Q2_TD_ColQ2-31_Meignie_Total_H-1144	ND	0	0	NA
	20170912_Q2_TD_ColQ2-31_Meignie_Total_A-1156	ND	0	0	NA
	20170912_Q2_TD_ColQ2-31_Meignie_Total_T-1120	*Macaca mulatta* polyomavirus 1	14	10	0.06
	20170912_Q2_TD_ColQ2-31_Meignie_Total_A-1189	Measles morbillivirus	217	217	1.01
	20170912_Q2_TD_ColQ2-31_Meignie_Total_T-1123	Measles morbillivirus	329	199	1.37
PXD030388 Peters et al. (2022) (45)	**04232021_Banfield_Phage_063B_300 kDa_FT_10uL_1D_PR180_QE1_s10**	crAssphage cr6_1	36	14	0.16
PXD001165 Wynne et al. (2014) (46)	Slice_9	Hendra henipavirus	11	11	0.31
	Slice_8_rpt	Hendra henipavirus	9	8	0.35
PXD002936 Dent et al. (2015) (47)	BeauR2	Avian coronavirus	60	43	1.74
	BeauR3	Avian coronavirus	63	38	2.43
PXD004095 Zhao et al. (2016) (48)	20140812_LC1_Sara_36h_MLAH_6-09	ND	0	0	NA
	20140812_LC1_Sara_36h_MLAH_6-10	Mastadenovirus sp.	10	3	0.16
PXD005104 Snijder et al. (2017) (49)	OR10_20151030_EC_HHV1_capsid_A	Human alphaherpesvirus 1	898	429	47.84
	OR10_20151030_EC_HHV1_UL17_02	Human alphaherpesvirus 2	252	107	55.88
PXD018594 Grenga et al. (2020) (50)	MS20-17_CoV2_J7MOI-01r	Severe acute respiratory syndrome-related coronavirus	300	58	1.03
	MS20-17_CoV2_J7MOI-001r	Severe acute respiratory syndrome-related coronavirus	298	56	1.20

TSMs and specific peptides (spePEP) numbers at the species taxonomical rank are indicated for every identified virus species. File names in bold highlight faeces samples collected from human subjects. Estimated biomass percentages describe the viral TSMs percentage compared to all TSMs. ND: not detected; NA: not applicable.

Mascot generic files, downloaded either directly or obtained by converting.raw files when unavailable, were subjected to the cascade search described above, in order to proteotype the organisms present in the samples, based on their experimentally acquired peptidomes.

As shown in Table 1, the following viral species were successfully identified: Influenza A virus, Respiratory syncytial virus, Human metapneumovirus, Norwalk virus, Rotavirus A, Human mastadenovirus F, Cowpox virus, Human mastadenovirus C, Zika virus, Measles morbillivirus, Severe acute respiratory syndrome-related coronavirus, Zika, Hendra henipavirus, avian coronavirus and the crAssphage cr6_1. Noteworthy, except the latter, all of these species present a clinical interest and their detection is highly relevant in a diagnostic context.

On top of the viral proteomics datasets that were assessed, a total of 47 samples from a dataset (PXD051723) - randomly selected after searching “cell culture” terms on the Pride platform - where no virus is expected have been analyzed in order to assess our method's specificity. Overall, the virus has been detected in 10 out of 47 samples, which account for a specificity of 79.6% if we add the two negative controls from the viral proteomics studies (Supplemental Table S2). Notably, our approach not only identified viruses but also provided insights into their production modes. For instance, discernible human signals, likely originating from HepG2 cells used during the virus production, were identified in the VACV_Cop_1 sample (Supplemental Table S3). In addition, signals from *Chlorocebus sabaeus*, associated with VERO cells, and *Bos taurus*, from the bovine serum albumin used for SARS-CoV-2 production, were detected in sample 201108OEc1_LD-2068-TNO-6A_017. Similarly, signals from *Macaca mulatta*, originating from LLC-MK2 cell cultures used in influenza virus production, were detected in the 220311OEc1_AP-2231-s5B-10uL--DDA90_018 sample.

As an example, detailed taxonomic inferences obtained during the different steps are further described in Supplemental Table S4 for the sample 150924QXc1_RB-0965-150922-004_011, referred to as sample 11, a fecal sample from a patient with a known gastrointestinal virus infection due to the Rotavirus A. In such a sample, many contributors, including bacteria, archaea, yeasts and fungi, residual food, the host, and viruses, can contribute to the proteomic signal (26). It is therefore a challenging sample for the identification of viruses per se. Taxon-Spectra Matches (TSMs) – as initially introduced in Pible *et al*. (2020) (27) – refer to Peptide-Spectra Matches assigned to different taxa. Hereafter, TSMs values are indicated after applying the parsimony principle, *i.e.* TSMs shared between taxa are assigned to the most abundant taxon. If we consider the superkingdom level, the 10,000 MS/MS spectra interpreted during step 1 resulted in 2132, 1619, 451, and 18 TSMs attributed to Bacteria, Eukaryota, Viruses, and Archaea, respectively. These TSMs indicated the presence of 35 genera, whose descendants were used to construct the database for Step 2. Then, the whole dataset, *i.e.* 38,707 MS/MS spectra, was queried, resulting in 4208, 2642, 877, and 0 TSMs assigned to Bacteria, Eukaryota, Viruses, and Archaea, respectively. Here, the number of validated genera was reduced to 32 as the search parameters are more stringent (*p* value 0.15) and the database is smaller, hence better corresponding to the real content of the sample. This search points toward 38 species that were included in the database used for step 3. Lastly, step 3 resulted in 4066 Bacteria-attributed TSMs over 22 species, 2569 Eukaryota-attributed TSMs shared between five species, and 840 Virus-attributed TSMs across two species. Two viral species were identified: the Rotavirus A with 834 TSMs and 191 specific peptides and the human rotavirus with 18 TSMs and five specific peptides.

**Fig. 2:**
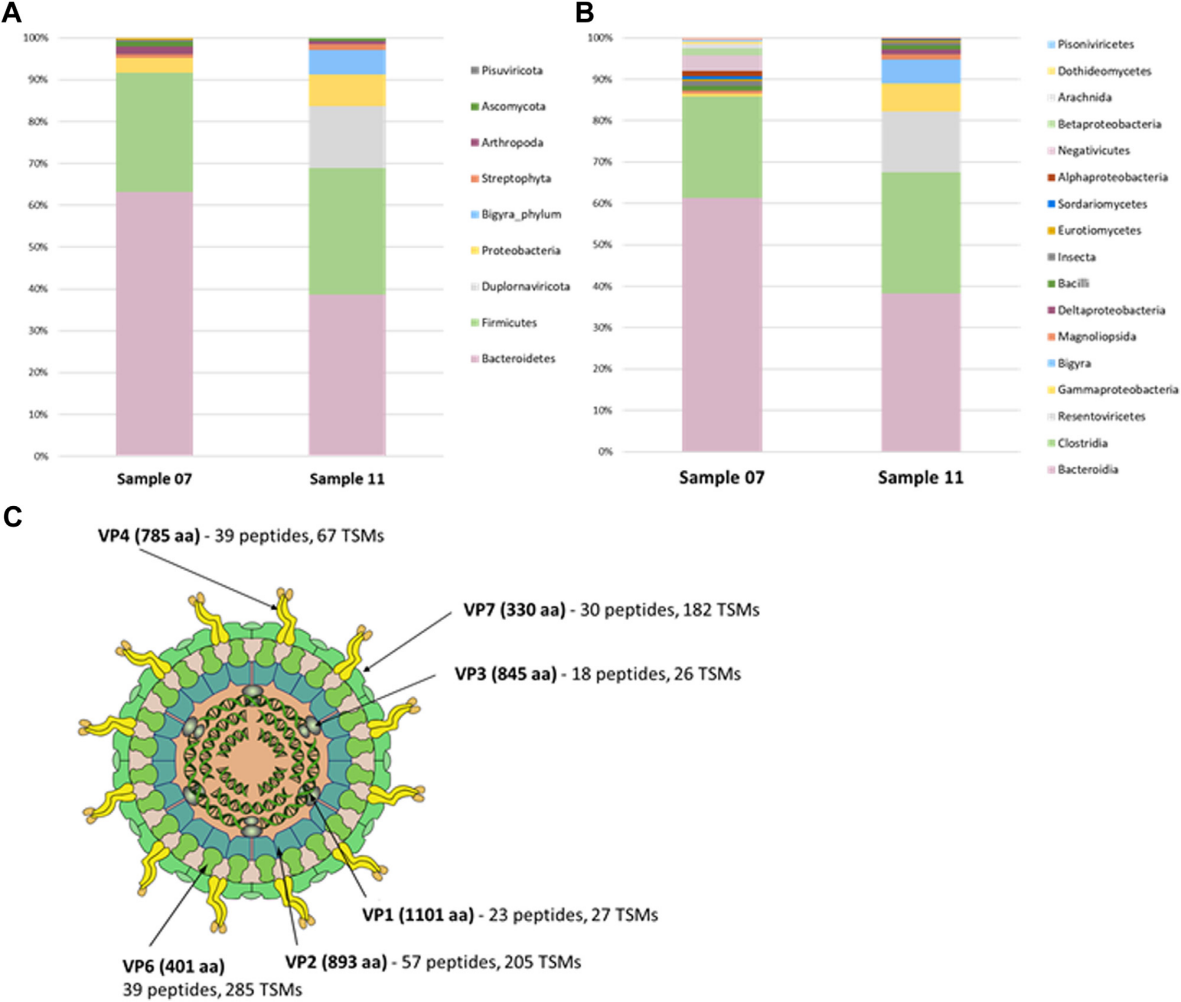
**Proteotyping of clinical samples harboring complex communities.** Taxonomical analysis of two feces samples at the phylum (*A*), class level (*B*) from the PXD036663 dataset (Balvers *et al*., 2023), and schematic representation of rotavirus A (credit: ViralZone, SIB Swiss Institute of Bioinformatics), detected in sample 11 highlighting the number of peptides, TSMs and length in amino acid (aa) for each structural protein (*C*).

Notably, our approach not only successfully identified the specific virus responsible for the patient's disease but also provided a comprehensive profile of the entire microbiota within the samples as illustrated by the taxonomical analysis conducted for the samples “150924QXc1_RB-0965-150922-002_007”, and “150924QXc1_RB-0965-150922-004_011”, referred to as sample 07 and sample 11, respectively (Fig. 2, *A* and *B*). While both samples mainly comprised Bacteroidetes, Firmicutes, and Chordata, commonly found in fecal samples (28), sample 11 harbored 5.8% of the total signal assigned to the *Bigyra* phylum, which, at the genus level, is attributed to *Blastocystis*, a human gut parasite. In addition, a total of 14.8% of TSMs were attributed to Rotavirus A, suggesting a high viral load in the patient and a complex clinical picture with several pathogens detected from the same sample. Structural proteins from the Rotavirus A, including VP4 and VP7 from the outer capsid, VP2 and VP6, from the intermediate capsid and VP1 and VP3 from the outer capsid, were identified with 39, 30, 57, 39, 23, and 18 peptides, respectively, as illustrated in Figure 2*C*.

If we focus on faeces samples analyzed with our methodology (Table 1), viral biomass ranged from 0.12 to 18.75%—based on all TSMs assigned at the species level - suggesting a wide diversity in the patient viral loads. As an example, signals attributed to Human mast adenovirus accounted for 3.86% and 18.75%, for two different patients, suggesting different viral loads for a similar species that could explain contrasted clinical symptoms. Similarly, signals attributed to Rotavirus A for two infected patients showed 2.51% and 11.18%, while both patients infected by the Norwalk virus displayed very few signals from viral origins, with 0.12% and 0.35%. Disparities in both structure and size among these viruses may account for these differences, as exemplified by Rotavirus A, which possesses six proteins and measures approximately 80 nm in width. In contrast, the Norwalk virus, with only two structural proteins, exhibits a narrower size of approximately 27 nm, resulting in a reduced amount of detectable material *via* mass spectrometry.

### Proteotyping of Saliva Spiked With Vaccinia Virus

**Fig. 3:**
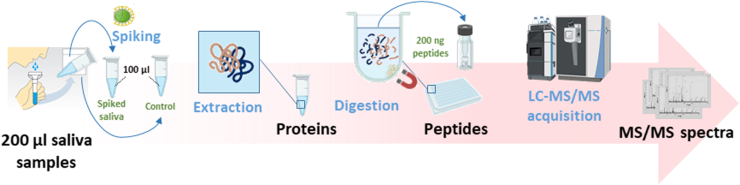
**Schematic representation of the sample preparation and data acquisition workflow.** Healthy donors were asked to spit saliva in tubes. Per donor, 100 μl of saliva were spiked with inactivated vaccinia virus and 100 μl were kept as control. Proteins were extracted followed by SP3 digestion in microplate. Peptides were quantified and 200 ng were injected on a LC-M/MS system.

Due to its non-invasive collection method, saliva proves to be an easily accessible fluid for the identification of viruses, particularly in the context of upper respiratory tract infections and viruses known to induce detectable viral loads in saliva, such as the Orthopoxviridae. Accordingly, our methodology was applied to saliva samples collected from four healthy donors, spiked with the vaccinia virus – a highly relevant model in the context of orthopox virus infection – at 5.10^6^ pfu/ml. Negative controls were conducted with the four unspiked salivae to ensure specificity. A rapid shotgun proteomics workflow including a 30-min nanoLC-MS/MS measurement was conducted (Fig. 3).

Noteworthy, the consistency and viscosity of the saliva were different between donors. Validated taxa and their number of TSMs at phylum and genus levels present in all saliva samples are presented in Supplemental Table S5. On average, the analyses resulted in the acquisition of 17,253 ± 1634 MS/MS spectra. As expected, the main signal was of human origin in every sample, accounting for 92.98 ± 6.95% of the total TSMs. The presence of the virus was validated in all samples with specific signal accounting from eight to 17 TSMs and from 7 to 14 specific peptides attributed to the orthopoxvirus genus within our database (Table 2). The peptides IGINYIIDTTSR, NIATSIYTIER, and QDFDNIIGVR belonged to the virion core protein P4a, and VVFAPPNIGYGR, from the virion core protein P4b, were detected in all positive samples and showed orthopox specificity. No false positive was detected in the four unspiked controls, emphasizing the specificity of the proteotyping-based detection method. In addition, the bacterium *Streptococcus pneumonia* was detected in Saliva C (asymptomatic donor) with 136 TSMs and 17 specific peptides, hence providing a double diagnosis thanks to our untargeted approach. In that case, signals from viral origin were not sufficient to discriminate the vaccinia virus as the detected proteins displayed more than 97% sequence similarity between multiple orthopox species, including cowpox, monkeypox, horsepox, and vaccinia virus, among others. A similar experiment conducted on saliva spiked with 1.10^7^ pfu/ml resulted in accurate species-level identification with 54 TSMs (Supplemental Table S6).

**Table 2:** Number of TSMs after parsimony and specific peptides attributed at the genus level detected in saliva samples spiked with vaccinia virus at 5.10^6^ pfu/ml

Sample	Saliva A	Saliva B	Saliva C	Saliva D
MS/MS spectra	14,259	17,495	16,572	16,758
*Homo sapiens* - TSMs	5409	6847	5805	6738
*Homo sapiens* - SpePeps	2974	3816	3248	3692
Orthopox - TSMs	8	8	17	9
Orthopox - SpePeps	8	7	14	9

### Proteotyping a Mixture of Three Phylogenetically Closely Related Viruses

In order to illustrate the method’s ability in discriminating phylogenetically closely related viruses within a single sample, a mixture containing three alphaviruses, namely, Pixuna virus, Rio Negro virus, and the Sindbis virus, was analyzed. Proteotyping yielded successful identification of the three species with 103, 78, and 45 TSMs, and 36, 29, and 22 specific peptides attributed to the Pixuna virus, Rio Negro virus, and the Sindbis virus, respectively (Supplemental Table S7). In parallel, we analyzed samples containing individual species. Our analysis confirmed that only the species present in each sample was detected, with no false positives observed. Specifically, Rio Negro virus, Sindbis virus, and Pixuna virus were detected with 176, 98, 183 TSMs and 46, 34, and 46 specific peptides.

## Discussion

The development of fully untargeted diagnostic techniques is required for a tailored and efficient therapeutic response. We have developed tandem mass spectrometry proteotyping to identify microorganisms, regardless of their origin (1, 29). We have shown its application for the detection of specific viruses, namely monkeypox virus and ancient lineages of coronaviruses (17, 24). Previous studies from other research groups have demonstrated the use of metaproteomics-derived workflow for diagnostic applications using customized databases including clinically significant taxa (30, 31). Pathogens from the upper respiratory tract such as SARS-CoV-2, *Streptococcus*, and *Klebsiella* species were successfully identified. However, the databases used until now are not comprehensive enough to ensure the taxonomic identification of multiple species from different superkingdoms in an untargeted fashion. Enlarging the search space to a database including any type of virus and all other possible organisms is necessary to permit a more universal identification procedure. Here, we demonstrated the power of a cascade search strategy - permitting a fully untargeted search - using all publicly available viral annotated proteomes as a database and proteomics datasets. This strategy enables the construction of a refined sample-specific database permitting the peptide-centric taxonomic identification of the organisms. It relies on a full sequence library of 36,223 viruses representing 25,169 distinct species and takes into account all the cellular organisms that could be present in the sample.

Recent developments in proteomics-based identification requiring no prior knowledge of the sample composition have emerged. Boulund *et al*. (2017) introduced TCUP, an approach designed to identify bacterial isolates and mixtures using discriminative peptides. However, its initial performance was limited as it used a database comprising 2785 bacterial genomes only (3). Similarly, MiCId conducts microbial identification based on a database that incorporates taxonomically attributed tryptic peptides (32). Later, an enhanced version that extends its capability to identify antibiotic resistance proteins was developed (33). TaxIt, a tool based on iterative queries using X!tandem performed accurate viral identification on public datasets (2). While proficient in offering strain level identification from simple samples with performances better than previous tools, its ability to detect viral signals from complex mixtures of clinical interest has not been yet documented. More recently, PepGM has emerged as a probabilistic graphical model tailored for strain-level virus taxonomic assignment. This tool operates by filtering out host and contaminant peptides before a search against the RefSeq Viral database and subsequent candidate taxa inference (13). While it showed good results for viral identification, this model has not been designed to identify multiple organisms within a sample. In addition, the filtering of host peptides could hamper virus detection by eliminating shared peptides between eukaryotic and viral species.

When compared to metagenomics approaches, our tandem mass spectrometry prototyping-based method presents several distinct advantages. Traditional metagenomics sequencing often requires targeted viral sequence capture, which limits the diagnostic capabilities and the range of detectable viruses (10, 34). Additionally, other studies highlighted the limitations of metagenomics in scenarios with low viral loads (35) and the lengthy run times involved (36). While these approaches showed great diagnostic potential and are easy to implement in clinical settings, proteins remain a better proxy when it comes to establishing relative microorganism biomass.

Here, our original and fully untargeted methodology permitted the identification of viruses present in samples from 53 different datasets. Among the nine files originating from the analysis of human fecal samples, seven led to viral identifications in accordance with the reported results from the original studies. Both unidentified samples carried the mamastrovirus one which was detected with the lowest number of peptides in the original study. Furthermore, our fully untargeted identification workflow depicted the comprehensive taxonomical insight of the sample, demonstrating its potential for clinical diagnosis. Noteworthy, our methodology displayed a sensitivity of 87.9% and a specificity of 79.6% with false positives that could be easily ruled out when taking into account the experimental context. However, its actual performance needs to be established in the future with a greater number of samples from a clinical cohort, to evaluate its diagnostic performances in a specific clinical context.

The presence of Orthopox virus was confirmed in saliva. Multiple Orthopox-specific peptides from structural proteins were detected, corroborating that our method can highlight the presence of a virus within a mixture containing other abundant signals. Noteworthy, discriminating minority signals in complex matrices can be challenging to overcome, even for next-generation sequencing approaches, due to the potential lack of amplification leading to false negative or sequencing artifacts (36).

The lowest viral TSMs percentage recorded in this study stood at 0.06%, and 0.12% in fecal samples. Virus detection could not be performed in two fecal samples due to their low abundance compared to the host and their microbiome. Sample fractionation prior to multiple LC-MS/MS runs or analyses with new generations of tandem mass spectrometers operating at high scan speed (200 Hz) offers great avenues for the improvement of the method's sensitivity (37). The efficacy of the proteotyping workflow described herein is anticipated to continually improve along with advancements in sample preparation, chromatography and mass spectrometry technologies. Progress in separative power, dynamic range, and acquisition speed of mass analyzers will enhance the detection of low amounts of specific peptides present in complex clinical matrices, as previously discussed (26) and illustrated by recent metaproteomics studies conducted with state-of-the-art equipments (38). Furthermore, future developments of sample preparation techniques, such as specific enrichment or predominant protein depletion could be beneficial for further improving the sensitivity and specificity of our methodology (39).

Overall, our workflow offers a distinctive advantage as it provides a singular approach capable of detecting any viral species in clinical samples provided that the species is encompassed in the queried database. Given that our current database includes 25,169 distinct viral species, a comprehensive coverage of all existing viruses is in principle obtained through the peptides that are conserved amongst closely related viruses. Even a new viral agent X (*e.g.* a new coronavirus) could be in principle identified as soon as it shares some peptide sequences with other viruses. In such a case, the result would be the identification at a higher taxonomical rank and the proposal of various species belonging to this branch of the tree of life, as previously explained (26). The detailed analysis of the protein sequence coverage would subsequently certify whether this constitutes a new strain. Here, the detection of the vaccinia virus was validated in clinically relevant concentrations (40), in a time and cost-efficient manner. This study, showcasing LC-MS/MS proteomics-based and comprehensive untargeted virus detection, has the potential to guide future advancements to meet the requirements of clinical diagnostic facilities.

## Data Availability

The mass spectrometry and proteomics dataset are available through the ProteomeX-change Consortium *via* the PRIDE partner repository (Available online: https://www.ebi.ac.uk/pride), under dataset identifier PXD050353 for the data treatments of the two examples detailed below issued from the public datasets and PXD050318 for the LC-MS/MS data experimentally acquired in our facility.

## Supplemental data

This article contains supplemental data.

## Conflict of interest

The authors declare no conflicts of interest with the contents of this article.
